# A high voltage gain solid-state transformer for integration of renewable energy and AC sources

**DOI:** 10.1038/s41598-024-77326-5

**Published:** 2024-10-26

**Authors:** Seyed Majid Hashemzadeh, Mohammed A. Al-Hitmi, Shirazul Islam, Atif Iqbal, Hadi Aghaei, Seyed Hossein Hosseini, Ebrahim Babaei

**Affiliations:** 1 Faculty of Electrical and Computer Engineering, University of Tabriz, Tabriz, Iran; 2 Department of Electrical Engineering, Qatar University, Qatar , Qatar; 3 Engineering Faculty, Near East University, Mersin 10, Nicosia, 99138 North Cyprus Turkey

**Keywords:** Solid state transformer, Photovoltaic, High voltage, Dual active bridge, High frequency transformer, Electrical and electronic engineering, Energy grids and networks

## Abstract

This paper introduces a novel high-voltage gain topology for a solid-state transformer, integrating a DC-DC converter and dual active bridge converters. The proposed design features three DC links operating at different voltage levels. The first DC link connects to a single-switch high step-up DC-DC converter, while the second DC link interfaces with an AC source via a rectifier, allowing the use of both DC and AC inputs. A high-frequency transformer ensures galvanic isolation between the sources and the third DC link. The DC-DC converter employs coupled inductors and voltage multiplier cells, offering distinct advantages such as a high voltage gain, reduced voltage stress on semiconductors, and minimized current ripple. These features make the topology highly suitable for transferring power from renewable energy sources, such as photovoltaic panels, to a high-voltage DC link in microgrid or nanogrid applications. The novelty lies in the combination of multiple voltage levels, high-frequency isolation, and the ability to handle both DC and AC inputs efficiently. An experimental prototype, delivering 620 W with a 25 V DC input and 110 V AC input, is built, and the results validate the converter’s effectiveness.

## Introduction

By harnessing solar power, solar photovoltaic (PV) system helps to reduce reliance on fossil fuels, mitigate climate change, and contribute to a cleaner and more sustainable energy combination^1,2^. The integration of PV systems into the electrical grid has gained significant momentum in recent years due to the growing emphasis on renewable energy sources^3–5^. As PV installations continue to expand, there is a need for efficient and reliable power conversion technologies to maximize the utilization of solar energy^6,7^. In this context, solid-state transformers (SSTs) offer promising solutions for PV system integration, enabling improved power quality, increased energy efficiency, and enhanced grid stability^8^. An SST for PV refers to the application of SST technology specifically tailored for photovoltaic power conversion and integration^9,10^. It serves as an interface between PV arrays and the grid, transforming and controlling the power generated by the solar panels to match the requirements of the grid or local loads^11,12^. Unlike traditional transformers, SSTs utilize advanced power electronic components and control algorithms to provide a range of functionalities and benefits for solar PV systems^13^. One of the key advantages of SSTs in solar PV applications is their ability to enhance power quality. SSTs can actively compensate for voltage fluctuations, harmonics, and other grid disturbances, ensuring stable and reliable power supply^14–16^. This capability is crucial for PV systems as they are subject to variations in solar irradiation and fluctuating power outputs^17^. By regulating voltage levels and compensating for power quality issues, SSTs enable solar PV systems to operate at maximum efficiency and minimize the impact on the grid^18^. Another significant advantage of SSTs in PV application is their ability to optimize energy utilization and increase overall system efficiency^19–21^. SSTs can employ advanced control algorithms to track the maximum power point of the PV panels, allowing for optimal energy extraction under varying solar conditions^22^. Additionally, SSTs can enable bidirectional power flow, facilitating grid interaction and energy storage integration, such as battery systems. This flexibility in power flow management contributes to higher energy conversion efficiency and improved system performance^23^. Moreover, SSTs for solar PV can enhance grid stability and enable the seamless integration of solar PV systems into existing distribution networks. By advanced control capabilities, SSTs can provide grid support functions such as reactive power compensation, voltage regulation, and fault detection^24,25^. These features help mitigate grid fluctuations caused by intermittent solar PV generation and ensure a smooth and reliable integration of solar PV source into the grid^26^. Furthermore, SSTs offer compact and lightweight designs, allowing for easier installation and reduced space requirements compared to traditional transformers^27^. This is particularly beneficial for PV installations where space constraints are often a concern. The compact size of SSTs also enables their integration within the PV system itself, reducing the need for additional external components and simplifying the overall system architecture^28^. In conclusion, SSTs for PV applications represent an innovative and efficient solution for integrating photovoltaic systems into the electrical grid. They provide enhanced power quality, increased energy efficiency, and improved grid stability^29^. As SST technology continues to advance, we can expect to see wider adoption of SSTs in PV installations, contributing to the continued growth and development of renewable energy^30^. Advanced semiconductor materials and power electronic components are used for.

building SST^31^. To regulate the flow of electric power, they use high-frequency switching devices such as insulated gate bipolar transistors (IGBTs) or silicon carbide (SiC) MOSFETs. In contrast to traditional transformers, which rely on magnetic cores and copper windings, SSTs employ power electronics to transform and regulate electrical energy^32^. The key advantages of SSTs are their superior control, flexibility, and efficiency as compared to traditional transformers. Following are a few highlights and advantages: *Voltage Regulation*: SSTs can actively regulate voltage levels, allowing for improved power flow management and optimization. This allows for more efficient use of renewable energy sources, such as solar and wind, whose output is frequently variable^33^. *Improved Power Quality*: SSTs may actively adjust for power quality concerns such as harmonics, voltage sags, and swells. SSTs may also offer reactive power assistance, which improves the stability of the grid and dependability. *Smart Grid integration*: Advanced communication and monitoring capabilities may also be added to SSTs. This facilitates real-time monitoring, fault detection, and grid management by allowing for smooth integration with smart grid systems^34^. *Compact and Lightweight Design*: SSTs have a lower footprint and weight than standard transformers, making them easier to install and move. This is especially useful in cities or for adapting existing electrical infrastructure^35^. *Energy Efficiency*: SSTs are more efficient than traditional transformers, particularly under fluctuating load situations. Their capacity to actively manage power flow and eliminate losses helps in reduction of total energy consumption^36^.

In this paper, utilizing DC-DC and dual active bridge converters, a novel high-voltage gain structure of an SST is presented. The proposed converter comprises three DC links with varying voltages. The first DC link is connected to a high voltage gain DC-DC converter, that offers benefits like high voltage gain, low peak voltage of diodes and power switch, and low input current ripple. This enables the reception of power from renewable energy sources like PV panels through a high voltage DC link. The second DC link is connected to an AC source through a rectifier, allowing for the utilization of both DC and AC sources. A HFT is employed to ensure galvanic isolation between the sources and the third DC link. The proposed topology is studied using analysis for operational modes, steady state behavior. Finally, an experimental prototype is built which is capable of delivering 620 W power to output load at a switching frequency of 50 kHz, and the experimental results are presented.

## Analysis of the proposed SST

**Fig. 1:**
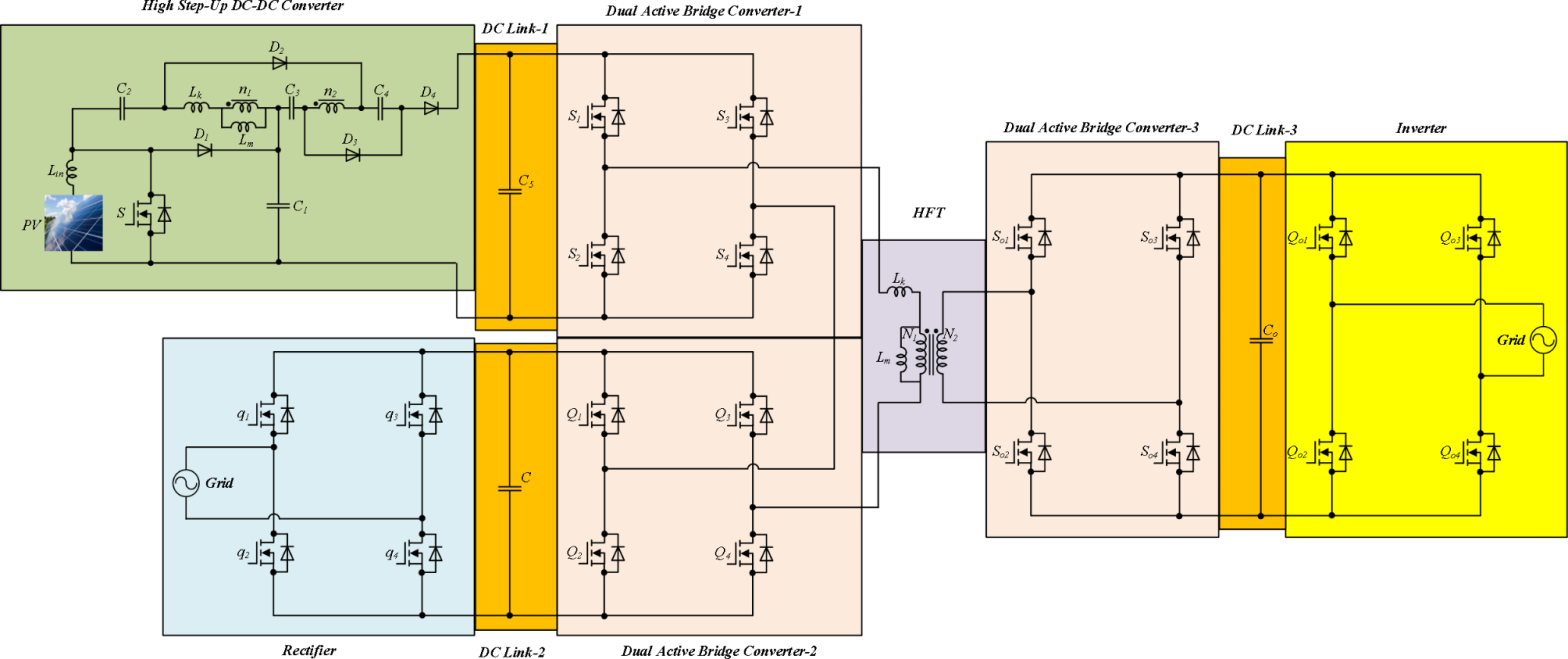
The proposed SST.

**Fig. 2:**
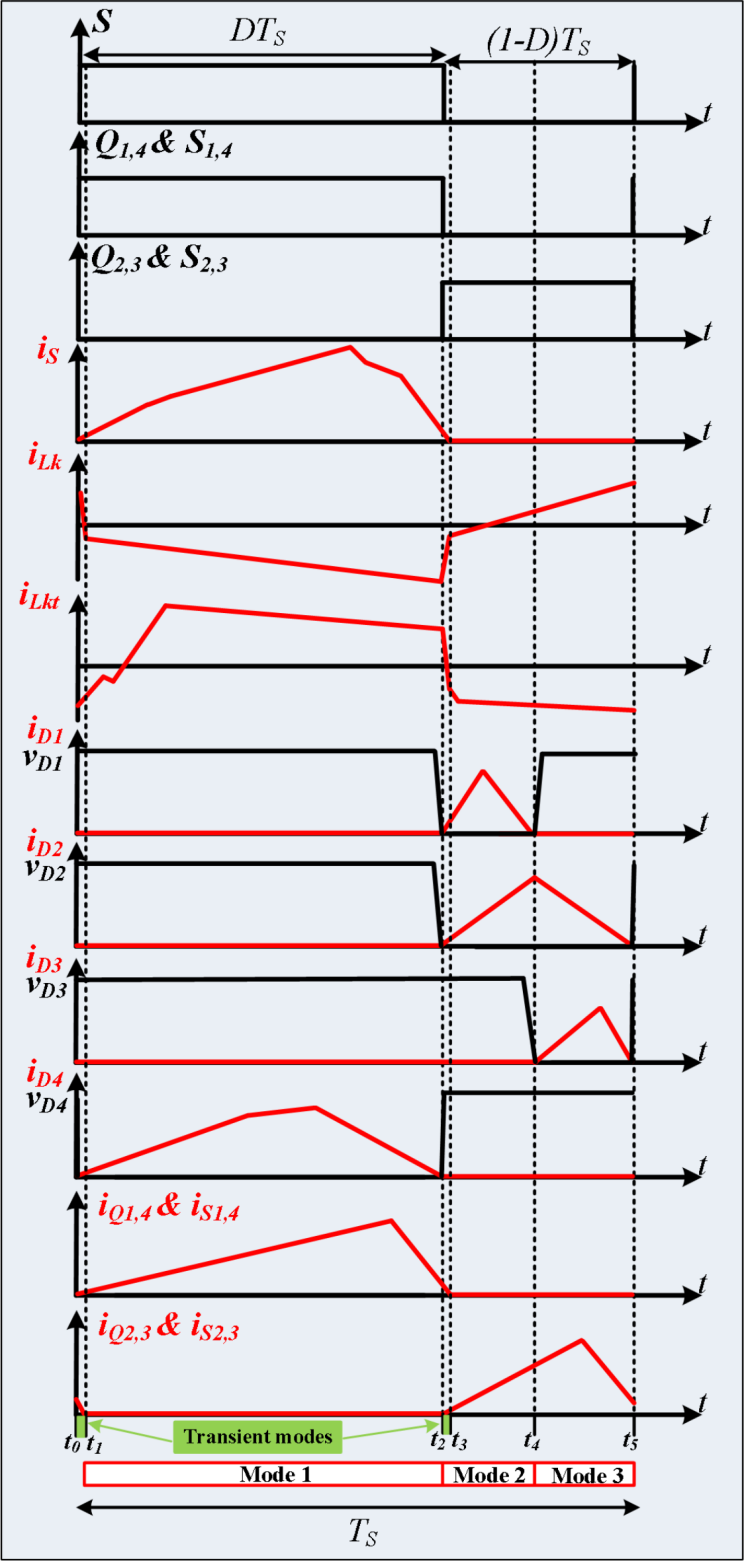
The main waveforms.

The proposed SST is depicted in Fig. 1. This converter includes two sections. The first section is DC-DC conversion, and the other section includes three dual active bridge (DAB) converters and a high frequency transformer (HFT). Also, the proposed converter has three DC links (DC link-1, DC link-2, and DC-link-3). The voltage of DC link-1 is obtained by boosting of a low voltage renewable energy source like PV panel. The voltage of DC link-2 is obtained by rectifying an AC voltage source. DC link-3 is used to provide the dc input voltage for an inverter which transfers energy to an AC load. The summation of output AC voltages of DAB converters is applied to the primary winding of the HFT. The DAB converter in the secondary side of the HFT can operate as a rectifier and obtain a DC voltage for DC link-3. Therefore, the are used for building SST^15^. To regulate the flow of electric power, they use high-frequency switching devices such as insulated gate bipolar transistors (IGBTs) or silicon carbide proposed SST provides two AC ports and one DC port, and, three DC link with different voltages. DC-DC conversion section includes one power switch (*S*), four diodes (*D*_*1*_, *D*_*2*_, *D*_*3*_, and *D*_*4*_), five capacitors (*C*_*1*_, *C*_*2*_, *C*_*3*_, *C*_*4*_, and *C*_*5*_), one input inductor (*L*_*in*_), and one coupled inductor. The turns ratio of the coupled inductor is defined as *n = n*_*2*_*/n*_*1*_. Each DAB converter has four switches. Also, the turns ratio of the HFT is presented as *N = N*_*2*_*/N*_*1*_. The PWM pulses of the power switches (*S*, *S*_*1*_, *S*_*2*_, *S*_*3*_, *S*_*4*_, *Q*_*1*_, *Q*_*2*_, *Q*_*3*_, *Q*_*4*_, *S*_*o1*_, *S*_*o2*_, *S*_*o3*_, and *S*_*o4*_), and main waveforms are depicted in Fig. 2. Based on this figure, the proposed SST operates in three main modes, and two transient modes. Because transient modes don’t affect steady-state analysis, only main operational modes are described in the subsequent subsections.

### Operation modes

#### Mode 1 [*t*_*0*_*< t < t*_*1*_

**Fig. 3:**
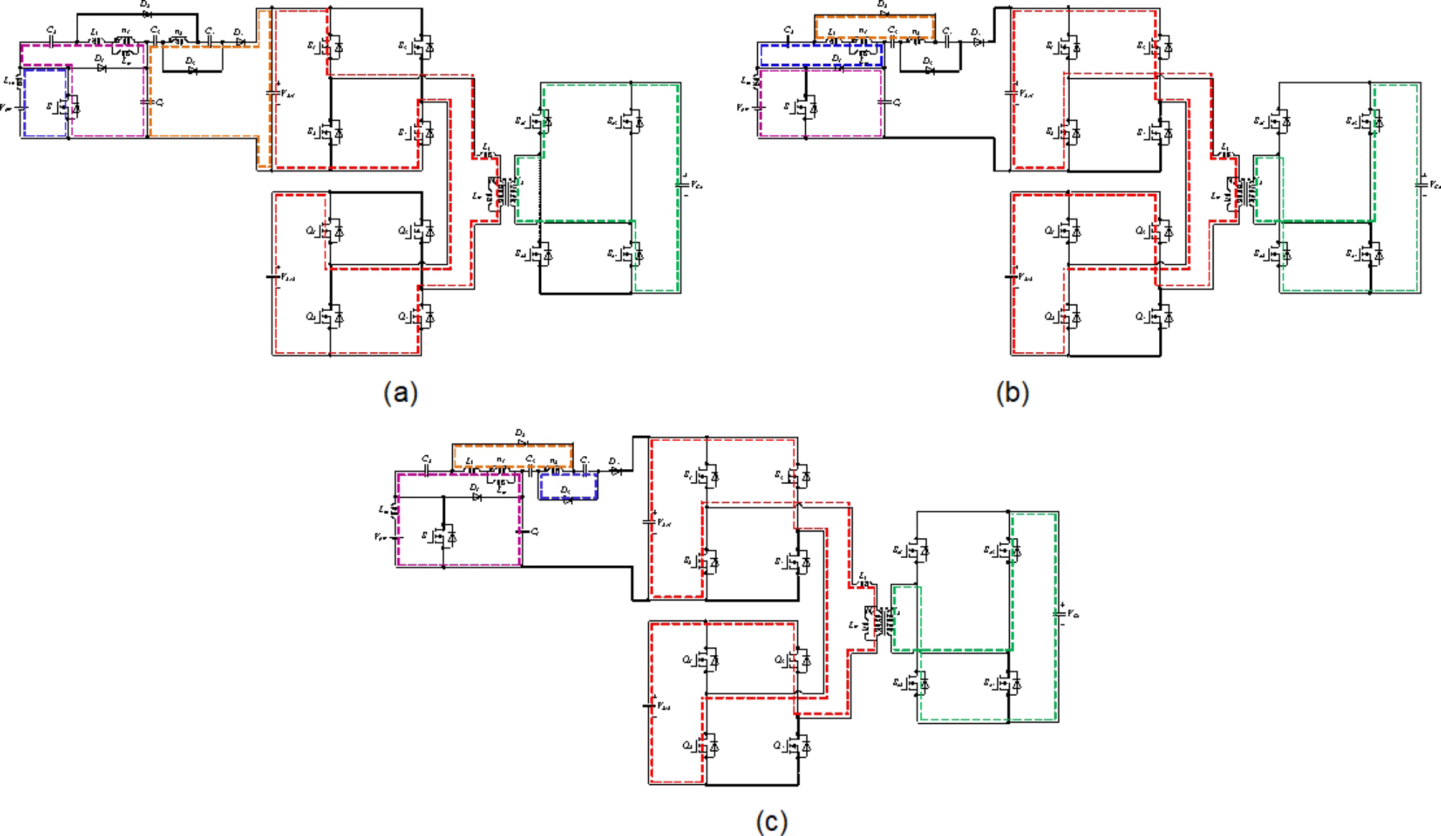
Operational modes, (**a**) mode 1, (**b**) mode 2, and (**c**) mode3.

This mode starts when power switches *S*, *S*_*1*_, *S*_*4*_, *Q*_*1*_, and *Q*_*4*_ are tuned on. Power switches *S*_*2*_, *S*_*3*_, *Q*_*2*_, and *Q*_*3*_ are turned off during mode 1. Furthermore, the DAB converter in the secondary side of HFT operates as a rectifier and all switches of this DAB are turned off, and the current flows through anti-parallel diodes of switches *S*_*o1*_~*S*_*o4*_. The equivalent circuit of mode 1 is illustrated in Fig. 3 (a). As noted from this figure, the currents flowing through the switches *S*_*1*_, *S*_*4*_, *Q*_*1*_, and *Q*_*4*_ are equal. The voltage of DC link-3 is *N*(*V*_*DC1*_+*V*_*DC2*_). This mode ends at time instant, *t = t*_*1*_, when diode *D*_*4*_ is reverse biased.

1 $${V_{{L_{in}}}}={V_{PV}}$$

2 $${V_{{L_m}}}=k\left( {{V_{{C_2}}} - {V_{{C_1}}}} \right)$$

3 $${V_{{C_5}}}={V_{{C_4}}}+{V_{{C_3}}} - nk{V_{{C_2}}} - \left( {1+nk} \right){V_{{C_1}}}$$

4 $${V_{{C_o}}}={V_{{N_2}}}=N{V_{{N_1}}}=N\left( {{V_{dc2}}+{V_{{C_5}}}} \right)$$

#### Mode 2 [*t*_*1*_*< t < t*_*2*_

At the start of mode 2, power switch *S* is turned off, and correspondingly, switches *S*_*2*_, *S*_*3*_, *Q*_*2*_, and *Q*_*3*_ are turned on. During this mode, *L*_*in*_ is discharged and its current is decreased. However, the voltage over *L*_*m*_ and *L*_*k*_ is positive and their currents are increasing. The standing voltages over switches *S*_*1*_ and *S*_*4*_ are equal to *V*_*C5*_*=V*_*DC1*_, and peak voltage across switches *Q*_*1*_ and *Q*_*4*_ are qual to *V*_*C*_*=V*_*DC2*_. The leakage energy of the coupled inductor is transferred to capacitor *C*_*3*_ through diode *D*_*2*_. Thus, the leakage energy is recycled during mode 2. The equivalent circuit of the converter during mode-2 is shown in Fig. 3 (b). Considering this figure, the following equations are obtained.

5 $${V_{{L_{in}}}}={V_{PV}} - {V_{{C_1}}}$$

6 $${V_{{L_m}}}=k{V_{{C_2}}}$$

7 $${V_{{C_3}}}=\left( {1+n} \right){V_{{L_m}}}=k\left( {1+n} \right){V_{{C_2}}}$$

8 $${V_{{C_o}}}= - {V_{{N_2}}}= - N{V_{{N_1}}}=N\left( {{V_{dc2}}+{V_{{C_5}}}} \right)$$

#### Mode 3 [*t*_*2*_*< t < t*_*3*_

At time instant, *t = t*_*2*_, diode *D*_*1*_ is turned off and diode *D*_*3*_ is forward biased. The state of other semiconductor switches is same as that in mode 2. Since, the diode *D*_*4*_ is tuned off, therefore, the energy of solar PV source is not delivered to DC link-3. However, the stored energy of capacitor *C*_*5*_ is transferred to output capacitor *C*_*o*_ through HFT windings. The configuration of this mode is depicted in Fig. 3 (c). During mode 3, the voltage across the capacitor *C*_*4*_ is as.

9 $${V_{{C_4}}}={V_{{n_2}}}=n{V_{{L_m}}}=\frac{n}{{1+n}}{V_{{C_3}}}$$

### Voltage calculations

This section presents the calculation of voltage across the semiconductor switches, diodes and capacitors. Using volt-sec balance principle on input inductors, *L*_*in*_ and *L*_*m*_, the expressions for *V*_*C1*_ and *V*_*C2*_ are obtained:

10 $${V_{{C_1}}}=\frac{1}{{1 - D}}{V_{PV}}$$

11 $${V_{{C_2}}}=D{V_{{C_1}}}=\frac{D}{{1 - D}}{V_{PV}}$$

Substituting the value of *V*_*c2*_ in (7), the expression for *V*_*C3*_ is

13 $${V_{{C_3}}}=\frac{{k\left( {1+n} \right)D}}{{1 - D}}{V_{PV}}$$

Also, substituting the value of *V*_*c3*_ in (9), the expression for *V*_*C4*_ is

14 $${V_{{C_4}}}=\frac{{knD}}{{1 - D}}{V_{PV}}$$

Substituting the values of *V*_*c1*_, *V*_*c2*_, *V*_*c3*_ and *V*_*c4*_ from (10), (11), (12) and (13) in (3), the simplified expression for *V*_*c5*_

15 $${V_{dc1}}={V_{{C_5}}}=\left( {\frac{{knD}}{{1 - D}}+\frac{{k\left( {1+n} \right)D}}{{1 - D}} - \frac{{knD}}{{1 - D}}+\frac{{\left( {1+nk} \right)}}{{1 - D}}} \right){V_{PV}}$$

The voltage gain of DC-DC conversion section can be written as:

16 $${M_{DC/DC}}=\frac{{{V_{{C_5}}}}}{{{V_{PV}}}}=\frac{{{V_{d{c_1}}}}}{{{V_{PV}}}}=\frac{{k\left( {1+n} \right)D+\left( {1+nk} \right)}}{{1 - D}}$$

Furthermore, the ideal voltage gain is as follows:

17 $${M_{DC/DC}}=\frac{{\left( {1+n} \right)\left( {1+D} \right)}}{{1 - D}}$$

The voltage of DC link-3 versus DC link-1 and DC link-2 can be expressed as:

18 $${V_{dc3}}={V_{{C_o}}}=N\left( {{V_{dc2}}+\frac{{k\left( {1+n} \right)D+\left( {1+nk} \right)}}{{1 - D}}{V_{PV}}} \right)$$

The maximum blocking voltage over diodes *D*_*1*_, *D*_*2*_, and *D*_*3*_ can be calculated during mode 1, when these diodes are reverse biased. The expressions for *V*_*d1*_, *V*_*d2*_ and *V*_*d3*_ are

19 $${V_{{D_1}}}={V_{{C_1}}}=\frac{1}{{1 - D}}{V_{PV}}$$

20 $${V_{{D_2}}}={V_{{C_3}}} - \left( {1+n} \right){V_{{L_m}}}=\frac{{k\left( {1+n} \right)}}{{1 - D}}{V_{PV}}$$

21 $${V_{{D_3}}}={V_{{C_4}}} - n{V_{{L_m}}}=kn{V_{{C_1}}}=\frac{{kn}}{{1 - D}}{V_{PV}}$$

Power switch, *S* and diode, *D*_*4*_ are turned off during mode 2. Thus, considering Fig. 3 (b), the maximum voltage across these elements can be obtained.

22 $${V_{{D_4}}}={V_{{C_5}}} - {V_{{C_4}}} - {V_{{C_3}}} - {V_{{C_1}}}+kn{V_{{C_2}}}=\frac{{kn}}{{1 - D}}{V_{PV}}$$

23 $${V_S}={V_{{C_1}}}=\frac{1}{{1 - D}}{V_{PV}}$$

**Fig. 4:**
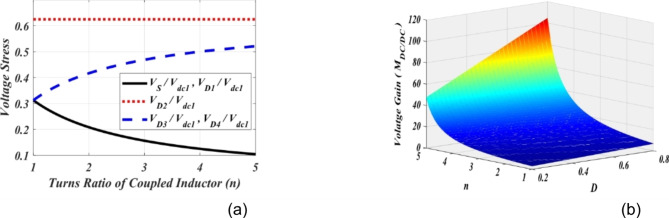
Voltage analysis, (**a**) normalized voltage stress (Eq. (24)) versus n and *D* = 0.6, and (**b**) The 3d view of the voltage gain *M*_*DC/DC*_.

The normalized voltage stresses of semiconductors are presented by (24). Additionally, the variation of voltage gain, *M*_*DC/DC*_ and voltage stresses across the switches and diodes versus coupled inductor turns ratio and duty cycle of power switch *S* are depicted in Fig. 4 (a) and (b). Plotting these figures, the voltage stresses across semiconductor switches and diodes in the DC-DC conversion section are normalized with respect to DC link-1 voltage (*V*_*dc1*_*=V*_*C5*_).

24 $$\left\{ \begin{gathered} \frac{{{V_S}}}{{{V_{dc1}}}}=\frac{{{V_{{D_1}}}}}{{{V_{dc1}}}}=\frac{1}{{\left( {1+n} \right)\left( {1+D} \right)}} \hfill \\ \frac{{{V_{{D_2}}}}}{{{V_{dc1}}}}=\frac{1}{{\left( {1+D} \right)}} \hfill \\ \frac{{{V_{{D_3}}}}}{{{V_{dc1}}}}=\frac{{{V_{{D_4}}}}}{{{V_{dc1}}}}=\frac{n}{{\left( {1+n} \right)\left( {1+D} \right)}} \hfill \\ \end{gathered} \right.$$

Finally, the voltage stresses of power switches included in DAB converters are calculated as (25).

25 $$\left\{ \begin{gathered} {V_{{S_{1\sim 4}}}}={V_{{C_5}}}=\frac{{\left( {1+n} \right)\left( {1+D} \right)}}{{1 - D}}{V_{PV}} \hfill \\ {V_{{Q_{1\sim 4}}}}={V_{dc2}} \hfill \\ {V_{{S_{o1\sim o4}}}}={V_{{C_o}}} \hfill \\ \end{gathered} \right.$$

### Current calculations

The average current of input inductor *L*_*in*_ can be obtained versus current of DC link-1:

26 $$I_{{{L_{in}}}}^{{avg}}={M_{DC/DC}}{I_{d{c_1}}}=\frac{{\left( {1+n} \right)\left( {1+D} \right)}}{{1 - D}}{I_{d{c_1}}}$$

The maximum value of current flowing through *L*_*in*_ is equal to algebraic sum of average current and half of peak-to-peak ripple:

27 $$I_{{{L_{in}}}}^{{\hbox{max} }}=I_{{{L_{in}}}}^{{avg}}+\frac{{\Delta {i_{{L_m}}}}}{2}=\frac{{\left( {1+n} \right)\left( {1+D} \right)}}{{1 - D}}{I_{d{c_1}}}+\frac{{D{V_{{L_{in}}}}}}{{2{f_s}{L_{in}}}}$$

The average currents flowing through the diodes in DC-DC conversion section are equal to *I*_*dc1*_. Based on the proposed SST’s configuration, the average current of power switch *S* is calculated as (28):

28 $$I_{S}^{{avg}}=I_{{{L_{in}}}}^{{avg}} - I_{{{D_1}}}^{{avg}}=\frac{{\left( {1+n} \right)\left( {1+D} \right)+\left( {D - 1} \right)}}{{1 - D}}{I_{d{c_1}}}$$

Therefore, considering the mean value, the peak current of switch *S* can be written as follows:

29 $$i_{S}^{{peak}}=\frac{{\left( {1+n} \right)\left( {1+D} \right)+\left( {D - 1} \right)}}{{D\left( {1 - D} \right)}}{I_{d{c_1}}}+\frac{{D{V_{{L_{in}}}}}}{{2{f_s}{L_{in}}}}$$

Considering the fact that, the average current of capacitors is zero, and using KCL, the average current of magnetizing inductance is obtained versus *I*_*s*_ and *I*_*Lin*_.

30 $$I_{{{L_m}}}^{{avg}}=I_{{{L_{in}}}}^{{avg}} - I_{S}^{{avg}}={I_{d{c_1}}}$$

Also, the maximum current of *L*_*m*_ is

31 $$I_{{{L_m}}}^{{\hbox{max} }}={I_{d{c_1}}}+\frac{{D{V_{{L_m}}}}}{{2{f_s}{L_m}}}$$

The peak current or current stress of diode *D*_*1*_ can be calculated during mode 2 operation, when the diode *D*_*1*_ is forward biased and is given by

32 $$i_{{{D_1}}}^{{peak}}=I_{{{L_{in}}}}^{{\hbox{max} }}+I_{{{L_m}}}^{{\hbox{max} }}=\frac{{\left( {1+n} \right)\left( {1+D} \right)+\left( {1 - D} \right)}}{{\left( {1 - D} \right)}}{I_{d{c_1}}}$$

The peak current of diodes *D*_*2*_, *D*_*3*_, and *D*_*4*_ are obtained as

33 $$i_{{{D_2}}}^{{peak}}=i_{{{D_3}}}^{{peak}}=\frac{{2{I_{d{c_1}}}}}{{1 - D}}$$

34 $$i_{{{D_4}}}^{{peak}}=\frac{{2{I_{d{c_1}}}}}{D}$$

## Design of the components

This section presents the design consideration of the used components which are semiconductors, capacitors, and magnetic devices.

## Semiconductors

The semiconductor switches and diodes of DC-DC conversion section and power switches included in DAB converters are selected based on maximum standing voltage and pathing current. These values are presented in the previous subsections of Sect. 2.

## Capacitors sizing

The values of capacitors are calculated based on this assumption that; the ripple voltage is almost 2% of average voltage across the capacitor. Using the voltage and current in the pervious section, the minimum values of capacitors are attained as follows:

35 $${C_1}=\frac{{D{I_{{C_1}}}}}{{{f_s}\Delta {V_{{C_1}}}}} \geqslant \frac{{D{I_{{C_1}}}}}{{2\% {f_s}{V_{{C_1}}}}}=\frac{{D\left( {1+n} \right)\left( {1+D} \right){I_{d{c_1}}}}}{{2\% {f_s}{V_{PV}}}}$$

36 $${C_2}=\frac{{D{I_{{C_2}}}}}{{{f_s}\Delta {V_{{C_2}}}}} \geqslant \frac{{D{I_{{C_2}}}}}{{2\% {f_s}{V_{{C_2}}}}}=\frac{{\left( {1+n} \right)\left( {1+D} \right){I_{d{c_1}}}}}{{2\% {f_s}{V_{PV}}}}$$

37 $${C_3}=\frac{{D{I_{{C_3}}}}}{{{f_s}\Delta {V_{{C_3}}}}} \geqslant \frac{{D{I_{{C_3}}}}}{{2\% {f_s}{V_{{C_3}}}}}=\frac{{{I_{d{c_1}}}}}{{1\% {f_s}\left( {1+n} \right){V_{PV}}}}$$

38 $${C_4}=\frac{{D{I_{{C_4}}}}}{{{f_s}\Delta {V_{{C_4}}}}} \geqslant \frac{{D{I_{{C_4}}}}}{{2\% {f_s}{V_{{C_4}}}}}=\frac{{{I_{d{c_1}}}}}{{1\% {f_s}n{V_{PV}}}}$$

39 $${C_5}=\frac{{D{I_{{C_5}}}}}{{{f_s}\Delta {V_{{C_5}}}}} \geqslant \frac{{D{I_{{C_5}}}}}{{2\% {f_s}{V_{{C_5}}}}}=\frac{{D\left( {1 - D} \right){I_{d{c_1}}}}}{{2\% {f_s}\left( {1+n} \right)\left( {1+D} \right){V_{PV}}}}$$

40 $$C=\frac{{D{I_C}}}{{{f_s}\Delta {V_C}}} \geqslant \frac{{D{I_C}}}{{2\% {f_s}{V_C}}}=\frac{{D{I_{d{c_1}}}}}{{1\% {f_s}{V_{d{c_2}}}}}$$

41 $${C_o}=\frac{{D{I_{{C_o}}}}}{{{f_s}\Delta {V_{{C_o}}}}} \geqslant \frac{{D{I_{{C_o}}}}}{{2\% {f_s}{V_{{C_o}}}}}=\frac{{D\left( {1 - D} \right){I_o}}}{{2\% {f_s}\left( {\left( {1 - D} \right){V_{d{c_2}}}+\left( {1+n} \right)\left( {1+D} \right){V_{PV}}} \right)}}$$

## Inductors sizing

To achieve continuous conduction mode (CCM) of operation for DC-DC conversion section, which is suitable for renewable energy sources, the magnitude of ripples in inductor current is assumed to be lower than half of the average current. The minimum value of *L*_*in*_ and *L*_*m*_ is presented as (42) and (43).

42 $${L_{in}}=\frac{{D{V_{{L_{in}}}}}}{{{f_s}\Delta {i_{{L_{in}}}}}} \geqslant \frac{{D{V_{{L_{in}}}}}}{{2{f_s}I_{{{L_{in}}}}^{{avg}}}}=\frac{{{D^2}{V_{PV}}}}{{2{f_s}\left( {1+n} \right)\left( {1+D} \right){I_{d{c_1}}}}}$$

43 $${L_m}=\frac{{D{V_{{L_m}}}}}{{{f_s}\Delta {i_{{L_m}}}}} \geqslant \frac{{D{V_{{L_m}}}}}{{2{f_s}I_{{{L_m}}}^{{avg}}}}=\frac{{{D^2}{V_{PV}}}}{{2{f_s}\left( {1 - D} \right){I_{d{c_1}}}}}$$

The turns ratio of the coupled inductor (*n = n*_*2*_*/n*_*1*_) can be calculated based on Eq. (44):

44 $$n=\frac{{\left( {\frac{{{V_{d{c_1}}}}}{{{V_{PV}}}}} \right)\left( {1 - D} \right) - \left( {1+kD} \right)}}{{k\left( {1+D} \right)}}$$

## High frequency transformer (HFT)

The HFT is selected based on the maximum voltage applied across the primary and secondary windings (*V*_*N1*_ and *V*_*N2*_), maximum pathing current of windings (*I*_*N1*_ and *I*_*N2*_), turns ratio (*N = N*_*2*_*/N*_*1*_), and switching frequency (*f*_*s*_) of the proposed SST.

## Efficiency analysis

The efficiency of the proposed SST is analyzed using calculation of elements losses. The total loss is presented in (45), which includes loss of power switches, diodes, capacitors, and magnetic devices.

45 $$\Delta P=\Delta {P_S}+\Delta {P_D}+\Delta {P_C}+\Delta {P_{Magnetics}}$$

In (45), $\Delta P$are the total loss, $\Delta {P_S}$, $\Delta {P_D}$, $\Delta {P_C}$, and $\Delta {P_{Magnetics}}$ power switches, diodes, capacitors, and magnetic devices losses.

Total Loss of power switches consists conduction and switching losses:

46 $$\Delta {P_S}=P_{{Loss}}^{S}+P_{{Loss}}^{{{S_{1 - 4}}}}+P_{{Loss}}^{{{Q_{1 - 4}}}}+P_{{Loss}}^{{{S_{o1 - o4}}}}$$

$P_{{Loss}}^{S}$, $P_{{Loss}}^{{{S_{1 - 4}}}}$, $P_{{Loss}}^{{{Q_{1 - 4}}}}$, and $P_{{Loss}}^{{{S_{o1 - o4}}}}$ are defined as (47):

47 $$\left\{ \begin{gathered} P_{{Loss}}^{S}={r_{on}}{\left( {I_{S}^{{RMS}}} \right)^2}+\frac{1}{2}{f_s}{V_S}I_{S}^{{avg}}({t_s}+{t_r}) \hfill \\ P_{{Loss}}^{{{S_{1 - 4}}}}={r_{on}}{\left( {I_{{{S_{1 - 4}}}}^{{RMS}}} \right)^2}+\frac{1}{2}{f_s}{V_{{S_{1 - 4}}}}I_{{{S_{1 - 4}}}}^{{avg}}({t_s}+{t_r}) \hfill \\ P_{{Loss}}^{{{Q_{1 - 4}}}}={r_{on}}{\left( {I_{{{Q_{1 - 4}}}}^{{RMS}}} \right)^2}+\frac{1}{2}{f_s}{V_{{Q_{1 - 4}}}}I_{{{Q_{1 - 4}}}}^{{avg}}({t_s}+{t_r}) \hfill \\ P_{{Loss}}^{{{S_{o1 - o4}}}}={r_{on}}{\left( {I_{{{S_{o1 - o4}}}}^{{RMS}}} \right)^2}+\frac{1}{2}{f_s}{V_{{S_{o1 - o4}}}}I_{{{S_{o1 - o4}}}}^{{avg}}({t_s}+{t_r}) \hfill \\ \end{gathered} \right.$$

In (47), ${r_{on}}$is the on-state resistance, *t*_*s*_ and *t*_*r*_ are the falling and raising times of the power switches.

Total Loss of diodes includes conduction and forward voltage losses:

48 $$\Delta {P_D}=\sum\limits_{{i=1}}^{4} {{r_{{D_i}}}{{\left( {I_{{{D_i}}}^{{RMS}}} \right)}^2}+{V_{{F_i}}}I_{{{D_i}}}^{{avg}}}$$

Where ${r_{{D_i}}}$is the internal resistance during on state, ${V_{{F_i}}}$is the forward voltage of didoes.

Furthermore, the total loss of capacitors and magnetic devices can be calculated using (49) and (50).

49 $$\Delta {P_C}={r_C}{\left( {I_{C}^{{RMS}}} \right)^2}+\sum\limits_{{i=1}}^{5} {{r_{{C_i}}}{{\left( {I_{{{C_i}}}^{{RMS}}} \right)}^2}}$$

50 $$\Delta {P_{Magnetics}}={r_{{L_{in}}}}{\left( {I_{{{L_{in}}}}^{{RMS}}} \right)^2}+\sum\limits_{{i=1}}^{2} {{r_{{n_i}}}{{\left( {I_{{{n_i}}}^{{RMS}}} \right)}^2}} +\sum\limits_{{i=1}}^{2} {{r_{{N_i}}}{{\left( {I_{{{N_i}}}^{{RMS}}} \right)}^2}}$$

In (49) and (50), ${r_C}$ and ${r_{{L_{in}}}}$ are the ESR o capacitors and inductors. The average and peak values of currents flowing through the semiconductors included in DAB converters, current of capacitors in each mode, and RMS values of currents of all components, which are essential to calculate the power efficiency are listed in Table 1.

## Experimental results

This section presents the experimental results of the proposed converter to verify the theoretical analysis. For this purpose, a prototype is built with 620 W output power. The parameters of the proposed converter are listed in Table 2. The presented topology can deliver power from two diverse power sources. One DC power source with voltage of 25 V is connected to the DC-DC converter. The coupled inductor turns ratio and duty cycle of power switch *S* is chosen as *N* = 1 and *D* = 0.6 to obtain an output voltage of 200 V at DC link-1. The DC-DC converter delivers a power of 200 W to DC link-1. Furthermore, an AC source with peak voltage of 110 V deliver 420 W to DC link-2 through a rectifier. After rectifying AC voltage, a 110 V DC voltage is obtained over DC link-2. Based on the configuration of the proposed converter, the summation of voltages of DC link-1 and 2, which is equal to *V*_*N1*_=200 + 110 = 310 V is applied to the primary winding of the HFT. The turns ratio of the HFT is 2:1. Thus, the voltage of secondary winding and also voltage of DC link-3 is equal to 155 V. The DC-DC converter includes 4 power diodes for which ultra-fast recovery STTH30R04 diode is used.

**Table 1:** The current equations.

Component	Current	Component	RMS Current
Switches of DAB converters	$$\left\{ \begin{gathered} I_{{{S_{1\& 4}}}}^{{avg}}=I_{{{Q_{1\& 4}}}}^{{avg}}=2D{I_{d{c_1}}} \hfill \\ I_{{{S_{2\& 3}}}}^{{avg}}=I_{{{Q_{2\& 3}}}}^{{avg}}=D{I_{d{c_1}}} \hfill \\ I_{{{S_{o1\& o4}}}}^{{avg}}=2ND{I_{d{c_1}}} \hfill \\ I_{{{S_{o2\& o3}}}}^{{avg}}=ND{I_{d{c_1}}} \hfill \\ \end{gathered} \right.$$	Switch *S*	$I_{S}^{{RMS}}=\frac{{2{f_s}{L_{in}}\left( {\left( {1+n} \right)\left( {1+D} \right)+\left( {D - 1} \right)} \right){I_{d{c_1}}}+{D^2}\left( {1 - D} \right){V_{{L_{in}}}}}}{{2{f_s}{L_{in}}\sqrt D \left( {1 - D} \right)}}$
		Diode *D*_*1*_	$I_{{{D_1}}}^{{RMS}}=\frac{{\left( {1+n} \right)\left( {1+D} \right)+\left( {1 - D} \right)}}{{\sqrt {1 - D} }}{I_{d{c_1}}}$
		Diodes *D*_*1*_ and *D*_*2*_	$I_{{{D_2}}}^{{RMS}}=I_{{{D_3}}}^{{RMS}}=\frac{{2{I_{d{c_1}}}}}{{\sqrt {1 - D} }}$
	$$\left\{ \begin{gathered} i_{{{S_{1\& 4}}}}^{{peak}}=i_{{{Q_{1\& 4}}}}^{{peak}}=2{I_{d{c_1}}} \hfill \\ i_{{{S_{2\& 3}}}}^{{peak}}=i_{{{Q_{2\& 3}}}}^{{peak}}={I_{d{c_1}}} \hfill \\ i_{{{S_{o1\& o4}}}}^{{peak}}=2 N{I_{d{c_1}}} \hfill \\ i_{{{S_{o2\& o3}}}}^{{peak}}=N{I_{d{c_1}}} \hfill \\ \end{gathered} \right.$$	Diode *D*_*4*_	$I_{{{D_4}}}^{{RMS}}=\frac{{2{I_{d{c_1}}}}}{{\sqrt D }}$
		Capacitor *C*_*1*_	$I_{{{C_1}}}^{{RMS}}={I_{d{c_1}}}\sqrt {\frac{{\left( {1 - D} \right){{\left(D {\left( {1+D} \right)\left( {1+n} \right)} -2\right)}^2}+{{\left( {D\left( {1+D} \right)\left( {1 + n} \right)} \right)}^2}}}{{D(1 - D)}}}$
		Capacitor *C*_*2*_	$I_{{{C_2}}}^{{RMS}}=\left( {\left( {1+n} \right)\left( {1+D} \right)} \right){I_{d{c_1}}}\sqrt {\frac{{1+D\left( {1 - D} \right)}}{{1 - D}}}$
Capacitor *C*	$${I_C}=\left\{ \begin{gathered} Mode1:{i_{{Q_1}}} \hfill \\ Mode2: - {i_{{Q_2}}} \hfill \\ Mode3: - {i_{{Q_2}}} \hfill \\ \end{gathered} \right.$$	Capacitors *C*_*3*_ & *C*_*4*_ and Secondary winding of coupled inductor	$I_{{{C_3}}}^{{RMS}}=I_{{{C_4}}}^{{RMS}}=I_{{{n_2}}}^{{RMS}}=2{I_{d{c_1}}}\sqrt {\frac{1}{{D\left( {1 - D} \right)}}}$
Capacitor *C*_*5*_	$${I_{{C_5}}}=\left\{ \begin{gathered} Mode1:{i_{{D_4}}} - {I_{d{c_1}}} \hfill \\ Mode2: - {I_{d{c_1}}} \hfill \\ Mode3: - {I_{d{c_1}}} \hfill \\ \end{gathered} \right.$$	Capacitor *C*_*5*_	$I_{{{C_5}}}^{{RMS}}={I_{d{c_1}}}\sqrt {\frac{{{{\left( {2 - D} \right)}^2}+D\left( {1 - D} \right)}}{D}}$
Capacitor *C*_*4*_	$${I_{{C_4}}}=\left\{ \begin{gathered} Mode1: - {i_{{D_4}}} \hfill \\ Mode2:0 \hfill \\ Mode3:{i_{{D_3}}} \hfill \\ \end{gathered} \right.$$	Primary winding of coupled inductor	$I_{{{n_1}}}^{{RMS}}={I_{d{c_1}}}\sqrt {\frac{{D\left( {1 - D} \right){{\left( {\left( {1+n} \right)\left( {1+D} \right)} \right)}^2}+{{\left( {n\left( {1+D} \right)+\left( {D - 1} \right)} \right)}^2}}}{{1 - D}}}$
Capacitor *C*_*3*_	$${I_{{C_3}}}=\left\{ \begin{gathered} Mode1: - {i_{{D_4}}} \hfill \\ Mode2:{i_{{D_2}}} \hfill \\ Mode3:{i_{{D_2}}}+{i_{{D_3}}} \hfill \\ \end{gathered} \right.$$	Capacitor *C* and Primary winding of HFT	$I_{C}^{{RMS}}=I_{{{N_1}}}^{{RMS}}={I_{d{c_1}}}\sqrt {1+3D}$
		Secondary winding of HFT	$I_{{{N_2}}}^{{RMS}}=N{I_{d{c_1}}}\sqrt {1+3D}$
Capacitor *C*_*2*_	$${I_{{C_2}}}=\left\{ \begin{gathered} Mode1:{i_S} - {i_{{L_{in}}}} \hfill \\ Mode2:{i_{{D_2}}} - {i_{{L_{in}}}} \hfill \\ Mode3: - {i_{{L_{in}}}} \hfill \\ \end{gathered} \right.$$		
		Switches of DAB converters	$$\left\{ \begin{gathered} I_{{{S_{1\& 4}}}}^{{RMS}}=I_{{{Q_{1\& 4}}}}^{{RMS}}=2\sqrt D {I_{d{c_1}}} \hfill \\ I_{{{S_{2\& 3}}}}^{{RMS}}=I_{{{Q_{2\& 3}}}}^{{RMS}}=\sqrt D {I_{d{c_1}}} \hfill \\ I_{{{S_{o1\& o4}}}}^{{RMS}}=N\sqrt D {I_{d{c_1}}} \hfill \\ I_{{{S_{o2\& o3}}}}^{{RMS}}=N\sqrt D {I_{d{c_1}}} \hfill \\ \end{gathered} \right.$$

**Table 2:** The value of the experimental prototype’s parameters.

Parameters	Value
PV DC Input	25 V
AC Input (Peak)	110 V
Frequency	50 kHz
Rated Power of DC-DC converter	200 W
Output Power	620 W
DC Link-1	200 V
DC Link-2	110 V
DC Link-3	155 V
Coupled Inductor Turns Ratio	1:1
HFT Turns Ratio	2:1 *V*_*N1*_=400 V:*V*_*N2*_=200 V
Duty cycles	Power Switch (S): *D* = 0.6
	Power Switches (*S*_*1*_-*S*_*4*_, *Q*_*1*_-*Q*_*4*_, *S*_*o1*_-*S*_*o4*_): *D* = 0.6

**Fig. 5:**
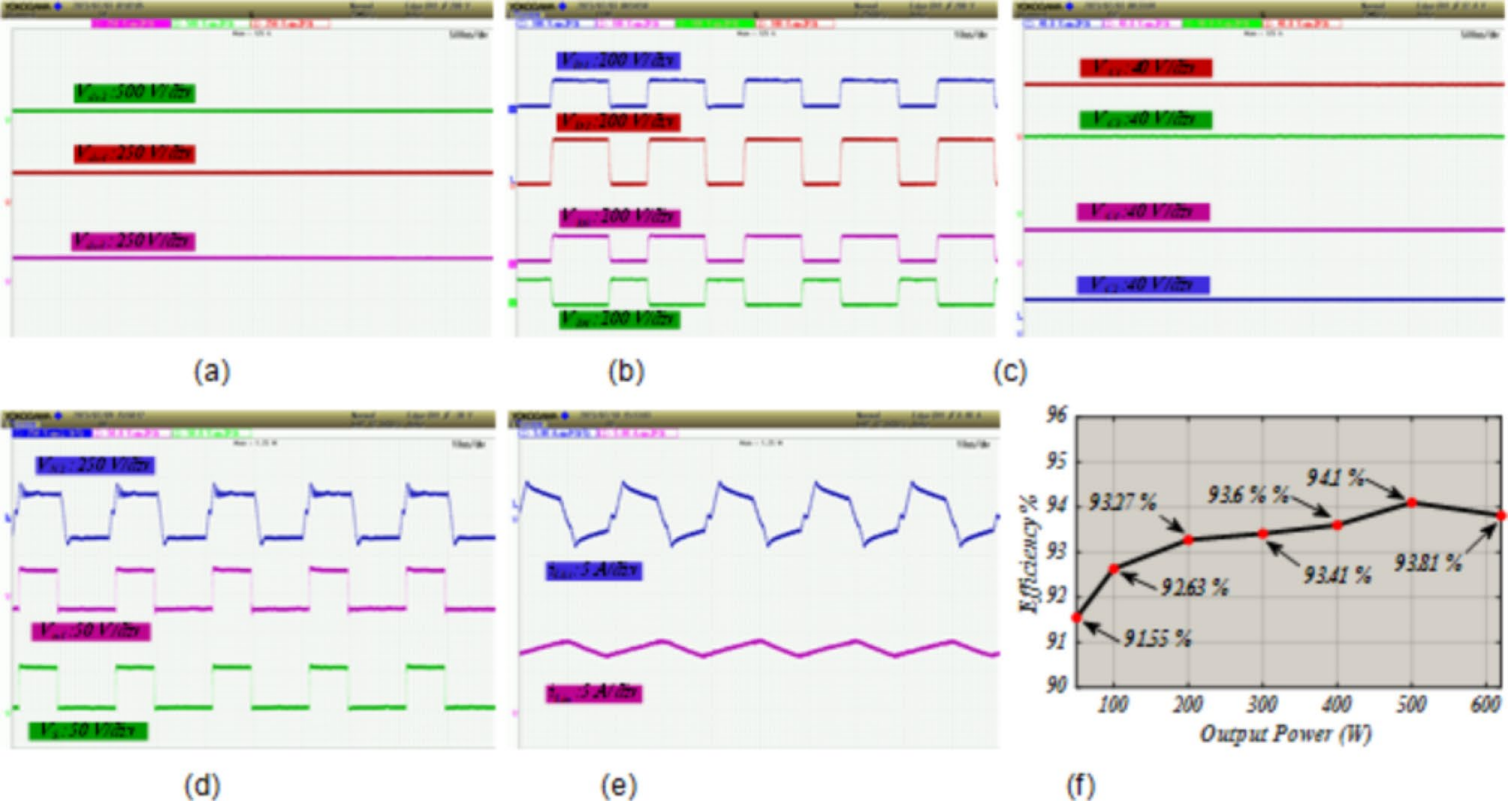
Experimental waveforms, (**a**) voltage of DC links, (**b**) voltage of diodes *D*_*1*_ ~ *D*_*4*_, (**c**), voltage of capacitors *C*_*1*_ *~ C*_*4*_, (**d**) voltage of power switch *S*, primary winding of the coupled inductor and secondary winding of the HFT, and (**e**) current waveform of input inductor *L*_*in*_ and primary winding of the HFT, and (**f**) the measured efficiency versus *P*_*o*_.

**Fig. 6:**
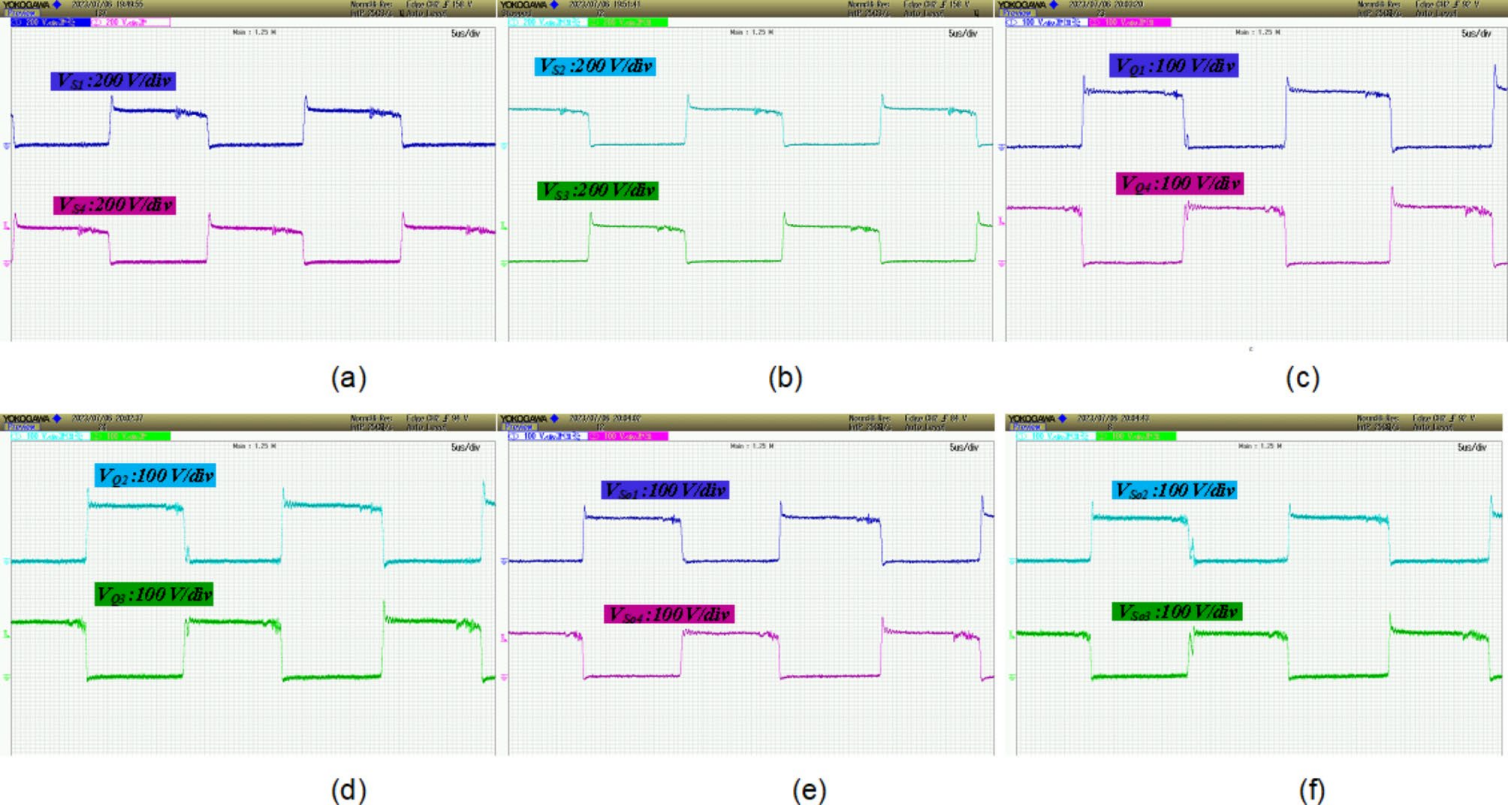
Experimental waveforms, (**a**) & (**b**) voltage of switches *S*_*1*_ *~ S*_*4*_, (**c**) & (**d**) voltage of switches *Q*_*1*_ *~ Q*_*4*_, (**e**) & (**f**) voltage of switches *S*_*o1*_*~S*_*o4*_.

**Fig. 7:**
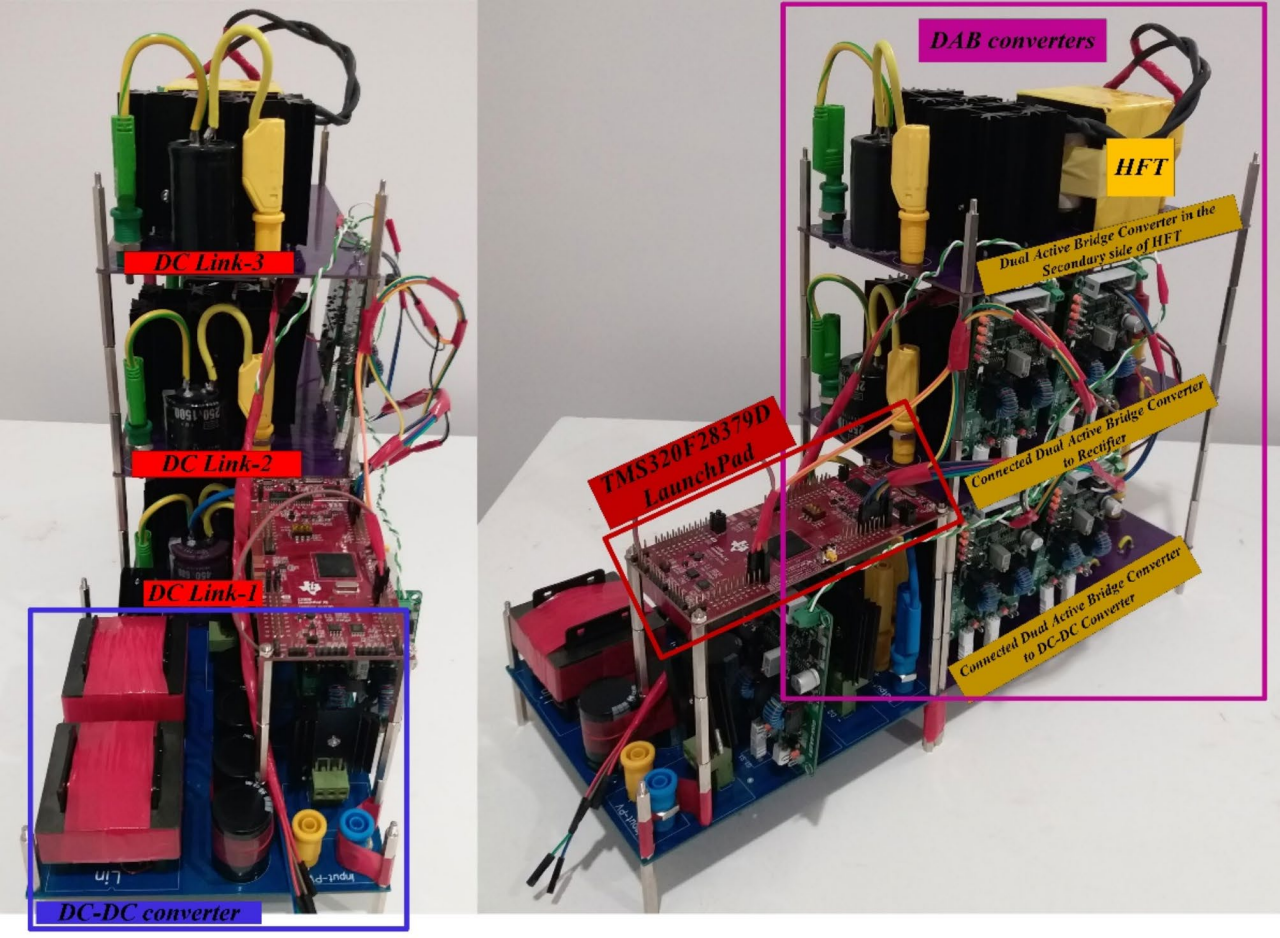
Experimental prototype.

**Fig. 8:**
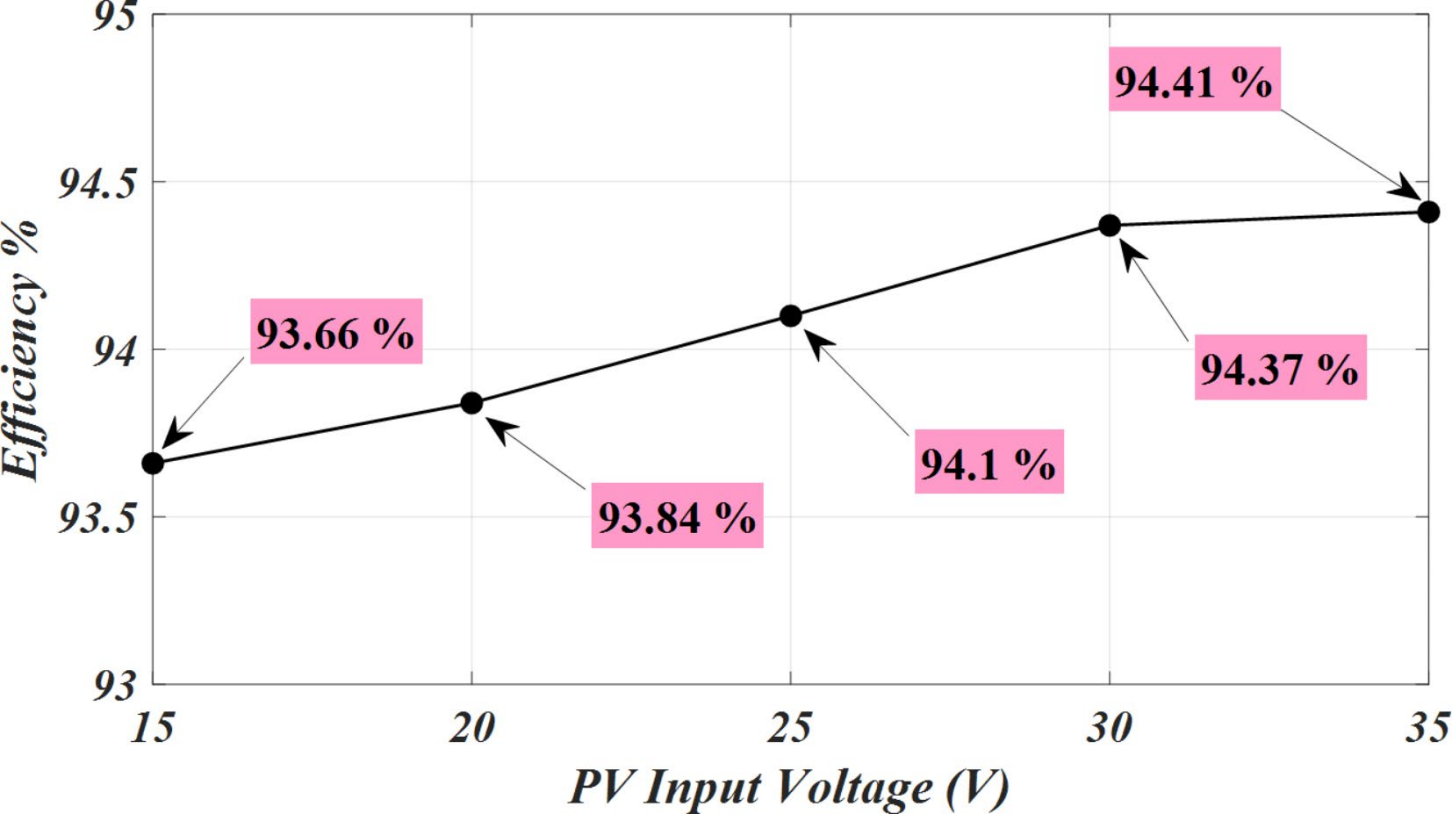
The efficiency curve versus PV input voltage.

**Fig. 9:**
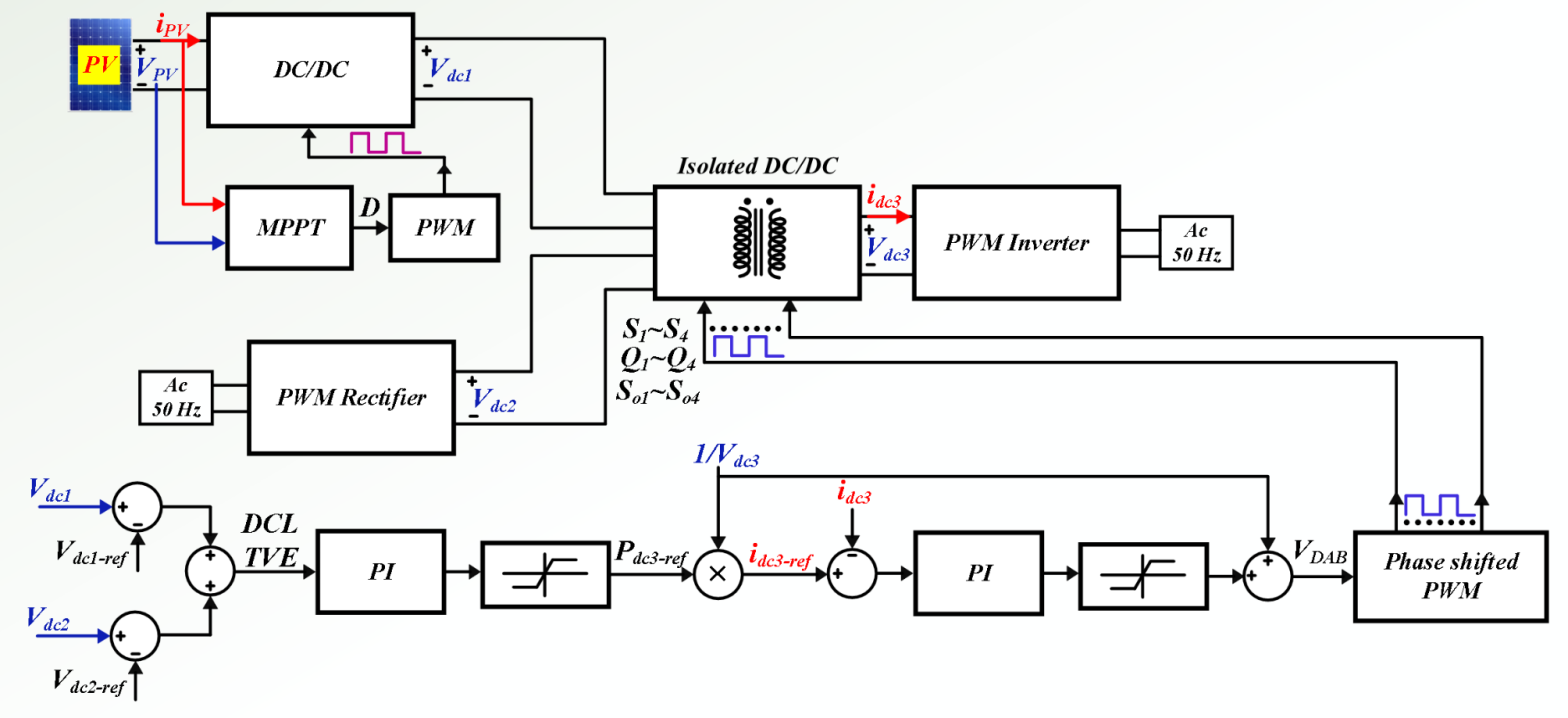
The proposed control method.

The power switch of DC-DC converter and power switches of the DAB converters are silicon carbide power MOSFET C3M0065090D. To generate PWM pules for power switches, TI C2000 TMS320F28379D launchpad is used. along with GDA-2A4S1 which is an isolated gate driver. Input value of inductance, *L*_*in*_ is selected to be 300 µH, while the magnetizing inductance, *L*_*m*_ of the coupled inductor is 300 µH, and its leakage inductance equal to 3 µH. To implement the input and coupled inductors, ferrite EE 55/28/21 cores are utilized. Also, all used capacitors are aluminum electrolytic, and the values of capacitances of capacitors *C*_*1*_ ~ *C*_*4*_ are330 µF each, while for capacitor C5 (DC Link-1) the selected value is 680 µF, capacitance of C (DC Link-2) is 1500 µF, and the capacitance value of output capacitor (DC Link-3), *C*_*o*_ is 470 µF. Figure 5 (a) shows the voltage waveforms of the DC link-1, 2 and 3. The voltages of DC link-1, 2, and 3 are measured as 192 V, 108 V, and 150 V, respectively. The waveforms of voltages across the diodes *D*_*1*_ ~ *D*_*4*_ are depicted in Fig. 5 (b). Voltage of diodes *D*_*1*_, *D*_*3*_, and *D*_*4*_ is almost 60 V, and voltage of diode *D*_*2*_ is measured as 119 V. These values validate the relations given by (19)-(22). Between diodes, the maximum normalized standing voltage is related to diode *D*_*2*_, which is 119 V/192 V = 0.61. Figure 5 (c) depicts voltages of capacitors *C*_*1*_ ~ *C*_*4*_. Voltage across the capacitor *C*_*1*_ is measured to be 54 V, and voltage of capacitors *C*_*2*_, *C*_*3*_, and *C*_*4*_ are obtained as 36 V, 70 V, and 35 V, respectively. These results confirm the mathematical equations presented in (10)-(14). Figure 5 (d) illustrate the voltage waveforms of the coupled inductor’s primary winding, HFT secondary winding, and power switch *S*. The maximum voltage appearing across windings *n*_1_ and *N*_2_ of the HFT are measured as 37 V and 150 V. The peak voltage across the power switch S is obtained to be 58 V. Therefore, the normalized voltage stress of this switch is equal to 58 V/ 119 V = 0.48. The input current of the DC-DC converter and leakage inductance of the HFT are shown in Fig. 5 (e). The average value of the input current *i*_*Lin*_ is obtained 8.4 A, and peak to peak value of this current is 1.8 A. This experimental result validates that due to low value of ripples in input current, the DC port is suitable for renewable sources. Due to the AC from, the average value of the pathing current from HFT is almost zero. Also, the maximum pathing current form primary side of the HFT is measured 4.5 A, and peak to peak value of this current is 8.5 A. The measured efficiency versus output power is presented in Fig. 5 (f). The efficiency of the proposed SST for variation of output power from 50 W to 620 W is obtained between 91.55% and 94.1%. The maximum efficiency is measured at *P*_*o*_= 500 W, which is 94.1%. Also, at the rated power, the efficiency achieved is 93.81%. The DAB converters in the primary side of the HFT act like inverters. The PWM pulses of each leg in these inverters are complimentary with a dead band equal to 1 µs. The DAB converter in the secondary side of the HFT used as a rectifier. Therefore, it doesn’t need PWM pulses, and current paths though anti-parallel diode of power switches. The voltage across the power switches related to these three DAB converters are depicted in Fig. 6 (a)- (f). According to these figures, the maximum voltage across power switches *S*_*1*_ ~ *S*_*4*_ is equal to 192 V. Also, *V*_*Q1*_~*V*_*Q4*_ are measured to be 108 V, and *V*_*So1*_~*V*_*So4*_ are obtained as 150 V. Furthermore, considering Fig. 7, the effect of leakage inductance of the HFT is visible, which make spikes on the voltage waveforms of power switch *S*_*1*_ ~ *S*_*4*_, *Q*_*1*_ ~ *Q*_*4*_, and *S*_*o1*_~*S*_*o4*_. The experimental prototype is shown in Fig. 8. To measure the efficiency versus PV input, the ac voltage and output load (500 W) are considered constant. Then for different voltage values of DC source, the efficiency is measured. For this work, the dc voltage is set from 15 V to 25 V. For a constant value of output load, it is clear that, the power loss is reduced with increasing the input voltage. Therefore, with increasing input dc voltage, efficiency is increased, which can be seen in Fig. 7. The proposed control strategy is shown in Fig. 9. As can be seen, MPPT of PV can be obtained by DC-DC converter inside of the SST. In fact, one of the reasons of using high step-up DC-DC converter in the proposed SST is to track the MPP of renewable energy sources like PV panel. The voltage of DC link 1 and 2 (*V*_*dc1*_ and *V*_*dc2*_) are regulated by dual active bridge converters in the primary side of the HFT. To control of voltages of DC link 1 and 2, the DC links total voltage error (DCL TVE) is summed and DCL TVE is controlled by PI controller. The controlled signal is considered as the reference output power of dual active bridge in the secondary side of the HFT. Thus, the injected current to the PWM inverter can be controlled. Furthermore, the voltage of DC link-3 can be regulated by PWM inverter. Totally, the voltage regulation cannot be an unsolvable problem using a three stages SST. It should be noticed that, three stages SST has DC links in both primary and secondary sides of the HFT, and the proposed SST can be categorized as a three stages SST.

**Fig. 10:**
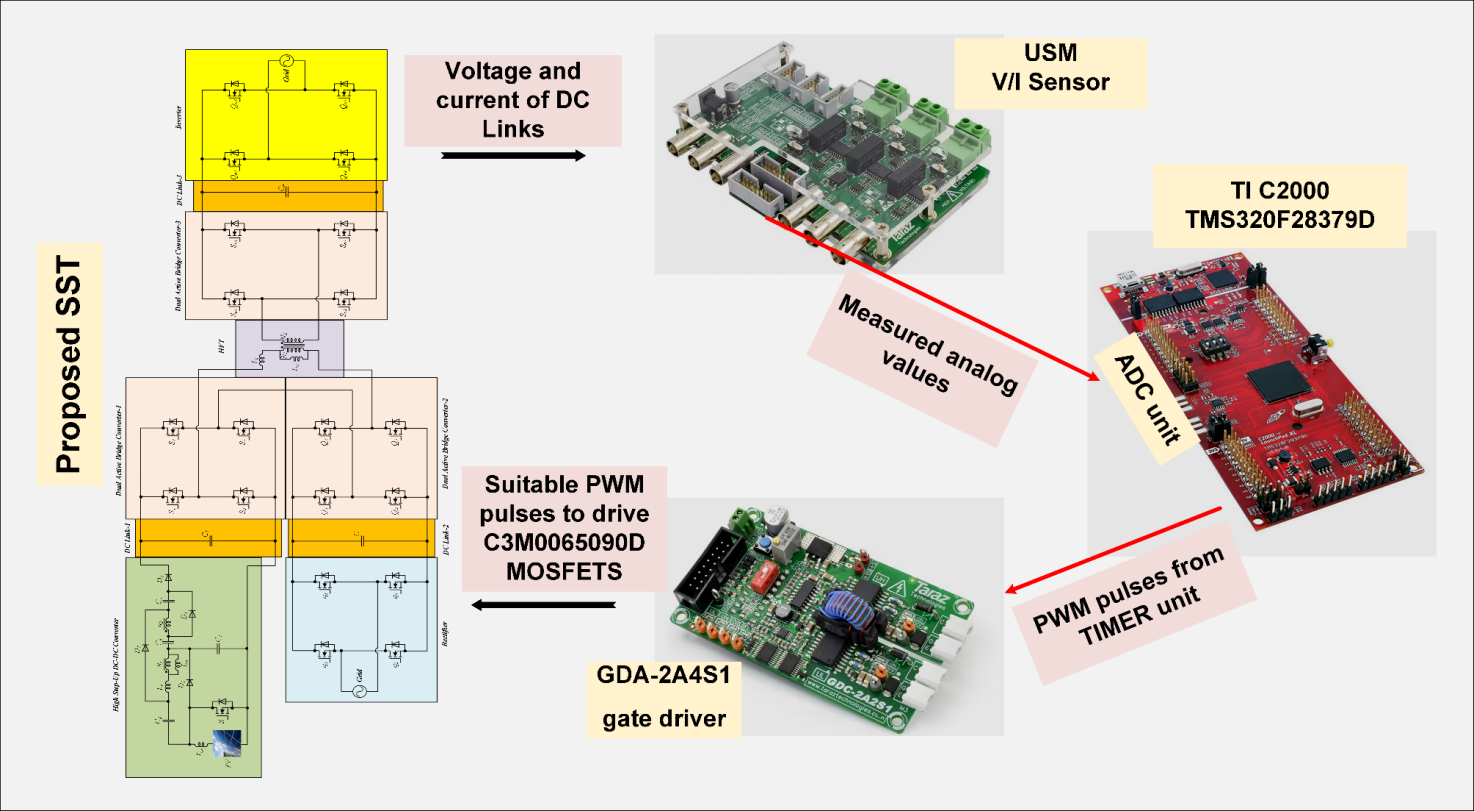
The experimental scheme for control of the proposed SST.

**Fig. 11:**
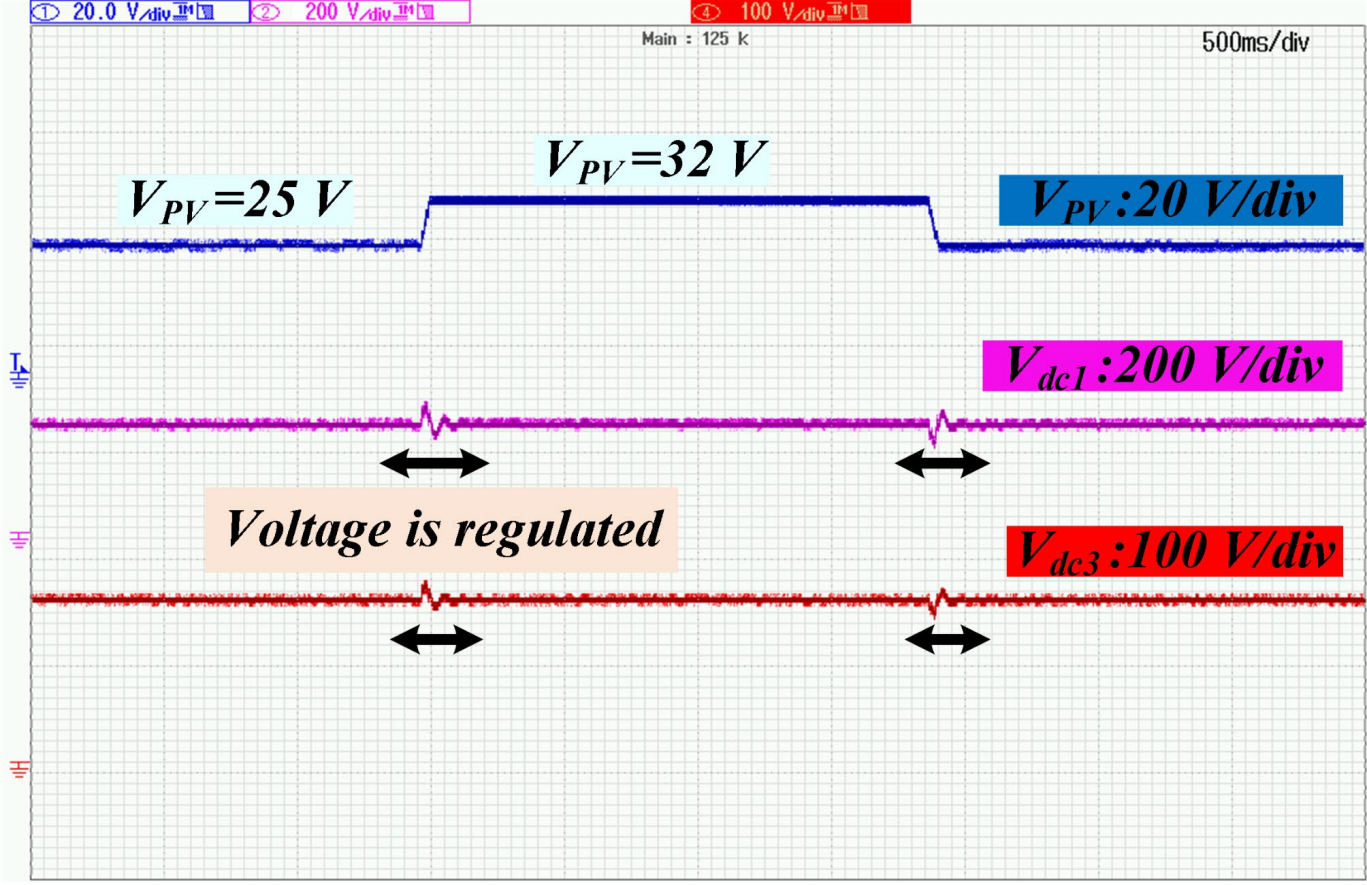
The closed-loop experimental waveforms.

Figure 10 illustrates the experimental control scheme for the solid-state transformer. In this scheme, the DC link voltages are regulated even when there are sudden changes in the PV and AC input sources. The corresponding experimental results are shown in Fig. 11, demonstrating the system’s performance under these varying input conditions. The proposed control method demonstrates the ability to effectively regulate the voltage of the DC links, even when there are fluctuations or variations in the input sources. This ensures stable performance and maintains the desired output despite changes in the input conditions, making the system more reliable and adaptable to dynamic operating environments. This capability is especially important for applications where input power may be inconsistent, such as in renewable energy systems, ensuring that the output remains steady and within the desired voltage range.

## Conclusion

In this article, a novel high-voltage gain topology of an SST is proposed. The converter presented consists of three DC links with different voltages. The first DC link is connected to a high voltage conversion ratio converter, offering advantages such as high voltage gain, low peak voltage of diodes and power switch, and low input current ripple. This allows power to be received from renewable energy sources like PV panels through a high voltage DC link. The second DC link is connected to an AC source via a rectifier, enabling the utilization of both DC and AC sources. Galvanic isolation between the sources and the third DC link is ensured through the use of an HFT. It should be noticed that, the third DC link is located in the output side of the converter. As a result, using HFT, the output port is isolated from input sources. The proposed topology is thoroughly surveyed by analysis of operational modes, steady state behavior, and efficiency Subsequently, an experimental prototype is built, capable of delivering 620 W of power to the output load at a switching frequency of 50 kHz, and the findings obtained from the experiments are showcased. The efficiency at rated power (*P*_*o*_=620 W) is 93.81%. Also, the maximum efficiency is obtained at *P*_*o*_= 500 W which is equal 94.10%.

## Data Availability

The data that support the findings of this study are available from the corresponding author upon reasonable request.
